# Editorial: Mental health in children and adolescents with a refugee background

**DOI:** 10.3389/fpsyg.2023.1284530

**Published:** 2023-09-15

**Authors:** Elisa Pfeiffer, Ilse Derluyn

**Affiliations:** ^1^Clinic for Child and Adolescent Psychiatry, Ulm University, Ulm, Germany; ^2^Department of Social Work and Social Pedagogy, Centre for the Social Study of Migration and Refugees, Ghent University, Ghent, Belgium

**Keywords:** children, adolescents, mental health, treatment, refugee

Numbers of children and adolescents who have to flee from their homes due various reasons such as war and conflict, economic instabilities, or consequences of climate change are increasing worldwide (https://www.unhcr.org/refugee-statistics/; July 25th, 2023). Host countries therefore need to have an answer on how to cater the needs of these individuals, both from a general health as a mental health perspective. A large and growing body of research conducted in different countries worldwide shows that children and adolescents with a refugee background report significantly higher rates of mental health disorders such as symptoms of posttraumatic stress disorder (PTSD), depression or anxiety (Blackmore et al., 2020), compared to children and adolescents without a refugee background (Spaas et al., 2022). It is therefore crucial to understand which factors (pre-, peri- and post-flight) impact their mental health (Scharpf et al., 2021) and to develop and evaluate treatment approaches that take these factors into account.

The present Research Topic aimed to shed further light on different aspects of the mental health and (trauma-focused) treatment of children and adolescents with a refugee background. The included ten research articles dispose of a large variety of methodological approaches, from network analysis to systematically implemented interviews, or from cross-sectional studies with large sample sizes to mixed-method treatment studies. The studies can be divided into three overarching themes: mental health and young refugees' concepts thereof, acculturation and integration, and interventions tailored to their specific needs.

In their cross-sectional analysis of *N* = 131 young refugees in Germany, Hornfeck et al. replicated prior studies as they found that 42% suffered from clinically relevant post-traumatic stress symptoms, 29% from depression and 21% from anxiety. Especially the number of reported traumatic events, daily stressors, and contact with family had a significant mental health impact. Potter et al. who conducted interviews with refugees in Germany further built on these findings as they found that severe physical abuse in childhood and the effects of the COVID-19-pandemic impacted refugees' emotional distress. In a study by Rizk et al. which investigated *N* = 52 Syrian adolescent refugees in South Beirut, the authors also documented high rates of mental health problems and identified several risky health concerns (e.g., lack of physical exercise or smoking) that could increase mental distress. Lastly, Behrendt et al. used a network approach to better understand the interplay between the mental health burden, past traumatic experiences and daily challenges. They found that especially daily stressors were associated with unaccompanied young refugees' mental health.

The role of daily stressors is also key in the second overarching theme in this issue, acculturation and integration. In their cross-sectional analysis of *N* = 132 young refugees in Germany, Garbade et al. found that the acculturation strategies “integration” and “assimilation” were most common in young refugees and that the orientation toward their home country was increased by higher daily stressors, but at the same time decreased by traumatic events in the past. Regarding young refugees' general acculturation process, Andersson and Overlien found in their interviews with *N* = 48 refugees who resettled in Norway that strategies such as gaining cultural competence or adapting and finding ways to contribute were named as especially helpful for their acculturation. The authors highlight the refugees' struggle in balancing between their original and host cultures, a theme also addressed in the study by Meyer et al. who investigated *N* = 101 accompanied Arabic-speaking refugee youth and found that their acculturation strategies, such as learning the local language and the number of friends in their new homes, were significantly associated with youth's mental health.

In sum, these studies report once again on the high rates of mental health challenges in this population and their significant association with the daily challenge to navigating through the new culture and society. Hence, two questions arise: (1) Why is mental health treatment uptake so low (Satinsky et al., 2019)? and (2) Which treatments and interventions do we need to address their specific needs in a trauma-informed framework? The study by Van de Meer et al. tried to answer the first question as they systematically interviewed young refugees on their concepts of somatic/mental illness and their mental health literacy. Not only did the interviewed young refugees report little knowledge of mental health (much lower compared to somatic illness), but none knew how to promote mental health. Hence, low-threshold services (maybe even implemented by peers) could be a means through which such populations could increase this knowledge and as such possibly increase their service uptake. Regarding the second question, two studies in this Research Topic describe promising refugee-specific approaches: The study by Van Es et al. evaluated a multi-model trauma-focused treatment approach, specifically designed for unaccompanied young refugees in the Netherlands. In their mixed-methods analysis, they could not find a significant symptom improvement, but in their interviews, all participants described the treatment approach as useful and felt it had positively impacted their wellbeing.

Since many trauma-focused treatments for refugees are supported by cultural mediators and interpreters; the study by Müller et al. is highly valuable as they developed and evaluated a specific training program for interpreters to support trauma-focused treatments. They found that their one-day training with *N* = 129 interpreters managed to not only help interpreters to gain knowledge but also to shift toward more treatment-appropriate attitudes.

The studies included in this Research Topic contribute to a better understanding of young refugees' mental health burden on the one hand, and to different aspects that need to be taken into account when treating their symptoms on the other hand. Future research needs to build on these findings by intensifying similar research in low-and middle-income countries and investigate children and adolescents who are still on the move. Preliminary studies on effective treatment approaches for young refugees (e.g., Pfeiffer et al., 2018; Unterhitzenberger et al., 2019) need to be investigated further and subsequently sustainably implemented and disseminated in countries which host large numbers of refugees (e.g., Iran, Türkiye or Colombia) but might not have the infrastructure to cater the refugees' needs yet.

## Author contributions

EP: Conceptualization, Writing—original draft, Writing—review and editing. ID: Conceptualization, Writing—original draft, Writing—review and editing.
